# Layer 2/3 Pyramidal Neurons of the Mouse Granular Retrosplenial Cortex and Their Innervation by Cortico-Cortical Axons

**DOI:** 10.3389/fncir.2020.576504

**Published:** 2020-11-03

**Authors:** Rita M. Robles, Eduardo Domínguez-Sala, Salvador Martínez, Emilio Geijo-Barrientos

**Affiliations:** ^1^Instituto de Neurociencias, Universidad Miguel Hernández—CSIC, Campus de San Juan, Alicante, Spain; ^2^CIBERSAM (Centro de Investigación Biomédica En Red en Salud Mental), Madrid, Spain

**Keywords:** late-spiking neurons, callosal axons, cortico-cortical axons, excitatory/inhibitory balance, neocortex, cortical circuits, synaptic mechanisms, retrosplenial cortex (RSC)

## Abstract

The retrosplenial cortex forms part of the cingulate cortex and is involved in memory and navigation. It is ventral region, the granular retrosplenial cortex, or GRSC is characterized by the presence, of small pyramidal neurons with a distinctive late-spiking (LS) firing pattern in layer 2/3. Using *in vitro* brain slices of the mouse GRSC we have studied the electrophysiological properties and synaptic responses of these LS neurons, comparing them with neighboring non-LS pyramidal neurons. LS and non-LS neurons showed different responses during cortical propagation of epileptiform discharges. All non-LS neurons generated large supra-threshold excitatory responses that generated bursts of action potentials. Contrastingly, the LS neurons showed small, and invariably subthreshold excitatory synaptic potentials. Although both types of pyramidal neurons were readily intermingled in the GRSC, we observed differences in their innervation by cortico-cortical axons. The application of glutamate to activate cortical neurons evoked synaptic responses in LS neurons only when applied at less than 250 μm, while in non-LS neurons we found synaptic responses when glutamate was applied at larger distances. Analysis of the synaptic responses evoked by long-range cortico-cortical axons (with the origin at 1200 μm from the recorded neurons or in the contralateral hemisphere) confirmed that non-LS neurons were strongly innervated by these axons, while they evoked only small responses or no response at all in the LS neurons (contralateral stimulation, non-LS: 194.0 ± 196.63 pA, *n* = 22; LS: 51.91 ± 35.26 pA, *n* = 10; *p* = 0.004). The excitatory/inhibitory balance was similar in both types of pyramidal neurons, but the latency of the EPSCs evoked by long-range cortico-cortical axons was longer in LS neurons (contralateral stimulation non-LS: 8.13 ± 1.23 ms, *n* = 17; LS: 10.76 ± 1.58 ms, *n* = 7; *p* = 0.004) suggesting a disynaptic mechanism. Our findings highlight the differential cortico-cortical axonal innervation of LS and non-LS pyramidal neurons, and that the two types of neurons are incorporated in different cortico-cortical neuronal circuits. This strongly suggests that the functional organization of the dorsal part of the GRSC is based on independent cortico-cortical circuits (among other elements).

## Introduction

The retrosplenial cortex (RSC) is the most caudal part of the cingulate cortex. The RSC is interconnected with areas of the brain (the hippocampal formation or anterior thalamic nucleus) that are important for memory formation, and a network of dorsal-medial cortical areas involved in spatial memory (Vann et al., 2009). In humans, the RSC plays a role in several cognitive functions, such as memory (Aggleton, 2014), spatial navigation (Epstein, 2008), orientation (Vann et al., 2009), and planning (Miller et al., 2014). In rodents, it comprises the entire posterior cingulate cortex (Vogt and Peters, 1982) and lesion studies have shown it is involved in spatial memory tasks (Sutherland et al., 1988), allocentric working memory tasks (Vann and Aggleton, 2002, 2004), and navigation (Cooper and Mizumori, 1999; Whishaw et al., 2001). The rodent RSC includes several cytoarchitectonic areas; according to Vogt et al. (2004) and Vogt (2014), these are 29a-c and 30. Areas 29a-c are located ventrally and correspond to the granular RSC (GRSC), while area 30 (or area 29d according to Vogt and Peters, 1982; see Sugar et al., 2011 for a review of the nomenclature of RSC cytoarchitectonic areas) is dorsal and corresponds to the dysgranular (or agranular) RSC.

Although we lack a detailed understanding of the contributions of each RSC sub-area, some authors have proposed that area 30 (dysgranular RSC) is important for the processing of visual information involved in allocentric spatial working memory (Vann and Aggleton, 2005), and area 29c (part of the GRSC) alone may contribute to spatial working memory processing (van Groen et al., 2004). These functional differences between dysgranular RSC and GRSC are presumably related to different connections with other cortical and subcortical areas such as the frontal, anterior cingulate, visual, and retro-hippocampal cortices, and the anterior thalamic nucleus (van Groen and Wyss, 1990, 1992, 2003; Shibata, 1998, 2000; Van Groen et al., 1993; Shibata and Naito, 2008). In addition to these extrinsic connections, distinct areas within the RSC also have complex interconnections (van Groen and Wyss, 1992; Van Groen et al., 1993; Jones et al., 2005; Shibata et al., 2009).

A distinctive feature of the rodent GRSC is the presence of an accentuated superficial layer 2/3, which is mainly formed by small pyramidal neurons with densely packed somata (Sripanidkulchai and Wyss, 1987; Ichinohe et al., 2008). These are callosal projection neurons, and in rats, their apical dendrites form noticeable bundles within layer 1 that are co-localized with patchy terminations of thalamocortical axons, mostly originated in the anterior thalamic nucleus, and with dendrites of parvalbumin-expressing interneurons (Sripanidkulchai and Wyss, 1986; van Groen and Wyss, 1990, 2003; reviewed in Ichinohe, 2012). A particularly remarkable feature of these neurons, described in the rat GRSC, is their late-spiking firing pattern, which is due to the presence of delayed rectifier and A-type potassium channels such as Kv1.1, Kv1.4, and Kv4.3 (Kurotani et al., 2013). This firing pattern could permit the integration of synaptic inputs with different timing, which is consistent with the GRSC’s suspected role in memory-related functions (Kurotani et al., 2013). Interestingly, the presence of late-spiking pyramidal neurons has been described in other brain areas related to the RSC such as the presubiculum (Abbasi and Kumar, 2013), the entorhinal cortex (Alonso and Klink, 1993), and the perirhinal cortex (Faulkner and Brown, 1999).

However, despite a large amount of information on the GRSC’s connectivity, very little is known about the role of GRSC late-spiking pyramidal neurons in the function of local and long-range cortical circuits. We have studied the electrophysiology and synaptic responses of mouse GRSC late-spiking pyramidal neurons and compared the results to those obtained in the neighboring, regular spiking pyramidal neurons, which are similar to those found in the dysgranular RSC layer 2/3 (Sempere-Ferràndez et al., 2018). Our results show that cortico-cortical axons originated in relatively distant areas (the contralateral homotopic cortex and ipsilateral dysgranular RSC) do not make direct excitatory contacts with late-spiking neurons, which only receive weak disynaptic responses of local origin (<250 μm from the soma). However, nearby pyramidal neurons that did not present a late spiking-firing received large synaptic contacts from both local and long-range cortico-cortical axons.

## Materials and Methods

### Animals and Ethical Approval

All experiments, except those recording epileptiform discharges, were done in GAD67:GFP mice; these animals are of the C57BL6 strain, present heterozygous GFP expression under the GAD67 promoter, and are usually referred to as GAD67:GFP (Tamamaki et al., 2003). By contrast, epileptiform discharges were recorded in C57BL6 mice. Mice were maintained, handled, and sacrificed following national and international laws and policies (Spanish Directive “Real Decreto 1201/2005”; European Community Council Directive 86/609/EEC). The experimental protocols were approved by the Experimental Research Ethics Committee of the Universidad Miguel Hernández.

### Slice Preparation

Brain slices of neocortex were prepared from male mice with postnatal age of 14–22 days. Animals were killed by cervical dislocation and their brains were quickly excised and submerged in ice-cold low Ca^2+^ / high Mg^2+^ cutting solution (composition in mM: NaCl 124, KCl 2.5, NaHCO_3_ 26, CaCl_2_ 0.5, MgCl_2_ 2, NaH_2_PO_4_ 1.25, glucose 10; pH 7.4 when saturated with 95% O_2_ + 5% CO_2_). Coronal slices (350 μm thick) were cut using a vibratome (Leica VT-1000; Germany), transferred to a glass beaker and submerged in artificial cerebrospinal fluid (ACSF; composition in mM: NaCl 124, KCl 2.5, NaHCO_3_ 26, CaCl_2_ 2, MgCl_2_ 1, NaH_2_PO_4_ 1.25, glucose 10; pH 7.4 when saturated with 95% O_2_ + 5% CO_2_) at 34°C for 30 min. The slices were stored submerged in ACSF for at least 1 h at room temperature before recordings were made. Slices were individually transferred to a submersion-type recording chamber and kept at 32–34°C during the recording period. The ACSF used to bath the slices was fed into the recording chamber at a rate of 2–3 ml·min^−1^ and was continuously bubbled with a gas mixture of 95% O_2_ + 5% CO_2_.

### Intracellular Recordings

We performed somatic whole-cell recordings from neurons whose somata were located in the dorsal part of layer 2/3 of the GRSC (−2.30 to −1.70 from Bregma). Pyramidal neurons were identified by their shape and confirmed by intracellular staining with Alexa Fluor 594 and the absence of GFP expression (in slices from GAD67:GFP mice). These neurons showed a characteristic pyramidal soma and a dominant apical spiny dendrite oriented to layer 1, while basal dendritic arbors were tangentially oriented.

Recordings were made under visual control using an upright microscope (Olympus BX50WI) equipped with Nomarski optics and a 40x water immersion lens. Measurements were obtained in current-clamp and/or voltage-clamp mode with a patch-clamp amplifier (Multiclamp 700B, Molecular Devices, San Jose, CA, USA). No correction was made for the pipette junction potential (which was estimated to be about −10 mV using the junction potential calculator included with the pClamp software). Voltage and current signals were filtered at 4 kHz and digitized at 20 kHz with a 16-bit resolution analog to digital converter (Digidata 1440A, Molecular Devices, San Jose, CA, USA). Clampex 10.3 software (part of the pClamp package; Molecular Devices, San Jose, CA, USA) was used to control stimulus generation and signal acquisition and analysis.

Patch pipettes made from borosilicate glass (1.5 mm o.d., 0.86 mm i.d., with inner filament) were used for intracellular recording; they had a resistance of 4–7 MΩ when filled with intracellular solution (composition in mM: K-gluconate 130, KCl 5, NaCl 5, EGTA 5, HEPES 10, Mg-ATP 2, Na-GTP 0.2, Alexa Fluor 594 0.01; pH 7.2 adjusted with KOH; 285–295 mOsm). The theoretical Nernst equilibrium potentials (in mV) for this K-based internal solution were: *E_K_* = −105.7, *E_Na_* = 89.3, *E_Cl_* = −68.5.

Current clamp recordings were performed at −70 mV and the neurons’ spontaneous resting membrane potential. Series resistance (R_s_) was measured and balanced on-line under visual inspection assisted by the Clampex software bridge balance tool. R_s_ was monitored at the beginning and end of each protocol and re-balanced if needed. Cells in which R_s_ was >40 MΩ were discarded (R_s_ was typically <25 MΩ). For voltage-clamp experiments, EPSCs (excitatory synaptic currents) and IPSCs (inhibitory synaptic currents) were recorded at holding potentials of −70 and 0 mV, respectively, values which are close to their respective reversal potentials. To hold the membrane at a specific membrane potential, the error in the membrane potential (V_e_) measurement was estimated from V_e_ = I_hold_ * R_s_, where I_hold_ is the holding current needed to set the holding potential. The holding potential was then corrected by adding the calculated V_e_ and holding the membrane to the desired V_hold_ (holding potential) while taking into account the error due to R_s_. Intrinsic membrane properties and synaptic responses were quantified using Clampfit 10.3.

### Electrical Stimulation

Electrical stimuli were applied using a concentric bipolar electrode (CBAFC75 outer diameter 125 μm, Frederick Haer and Co., Bowdoin, ME, USA) placed as indicated in the “Results” section. Concerning contralateral stimulation, we assessed the integrity of the callosal projection through extracellular recordings before the intracellular experiments (Sempere-Ferràndez et al., 2018). We used Single square current pulses of 0.1 ms and supra-maximal stimulus intensities applied at 0.2 Hz to evoke synaptic responses in the recorded neurons. To determine the supra-maximal strength, we first detected the stimulus strength evoking the maximal response by progressively increasing the stimulus; this strength was increased by 10% to establish the supra-maximal value. The range was 100–500 μA, but in most slices, it was 400–500 mA.

### Glutamate Application

Synaptic currents were evoked by the direct application of glutamate (1 mM glutamate dissolved in ACSF) using patch pipettes. The tip of the pipette was placed at several different distances from the soma of the recorded pyramidal neuron and glutamate was released by 20 ms pressure pulses of 5–10 psi applied with a Picospritzer (General Valve Corp. Fairfield, NJ, USA). Pressure pulses were applied at 20 s intervals.

### Intracellular Staining With Biocytin

Some neurons were labeled with biocytin using the method described by Marx et al. (2012). Briefly, biocytin was added to the intracellular solution to give a final concentration of 5 mg/ml. Slices containing stained neurons were fixed overnight at 4°C in 100 mM phosphate-buffered saline (PBS; pH 7.4) with 4% paraformaldehyde. They were rinsed several times in PBS containing 1% Triton X-100 and the endogenous peroxidase was blocked by incubation in 30% H_2_O_2_. The slices were then transferred to a complex of 1% avidin–biotinylated HRP with 0.5% Triton X-100 (ABC Peroxidase Standard PK-400 Vectastain; Vector Labs, Burlingame, CA, USA) and left for 1 h with gentle shaking. They were reacted using 3,3-diaminobenzidine (DAB; Sigma) and the reaction stopped by adding 30% H_2_O_2_. Finally, the slices were mounted on glass slides, embedded in glycerol jelly, and coverslipped. Biocytin-stained neurons were drawn using Neurolucida software (MBF Bioscience, Williston, VT, USA).

### Evoking and Recording Epileptiform Discharges

The slices were bathed in a modified ACSF (composition in mM: NaCl 124, KCl 5, PO_4_H_2_Na 1.25, MgCl_2_ 1, CaCl_2_ 1.2, NaCO_3_H 26, glucose 10; pH 7.4 when saturated with 95% O_2_ and 5% CO_2_). In modified ACSF and in the presence of 10 μM bicuculline (a GABA_A_ receptor antagonist) the stimulation of layer 1 evokes large oscillatory discharges, which were recorded extracellularly with patch pipettes filled with modified ACSF (Rovira and Geijo-Barrientos, 2016). Modified ACSF and bicuculline were used only in the experiments of propagation of epileptiform activity reported in Figure 4. In all other experiments, standard ACSF described above in the paragraph “slice preparation” was used.

**Figure 1:**
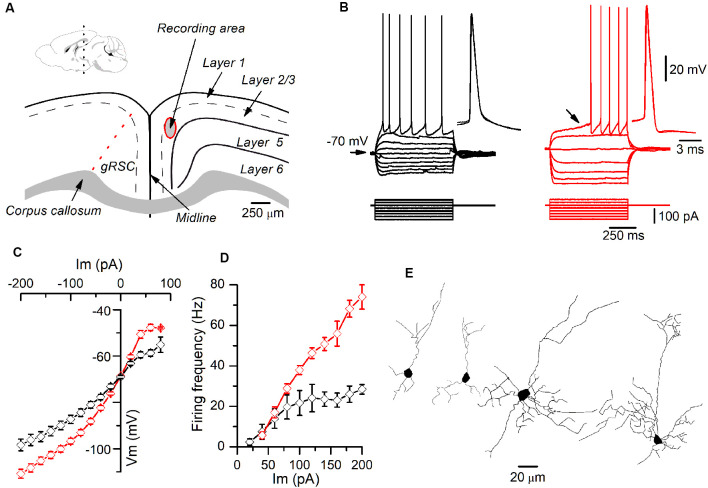
Different firing patterns of layer 2/3 pyramidal neurons recorded in the granular retrosplenial cortex (GRSC). **(A)** Coronal view of the mouse GRSC highlighting the recording area. The dotted red line shows the approximate limit between the GRSC and the dysgranular RSC; this limit has been set according to Vogt and Paxinos (2014). The inset shows the mid-sagittal mouse brain with the approximate level of the coronal slices used for recordings (black dotted line; drawing taken from https://portal.brain-map.org/, reference atlas version 1, 2008; available online at: http://connectivity.brain-map.org/; 2014 Allen Institute for Brain Science). **(B)** Responses to hyperpolarizing and depolarizing current pulses of two pyramidal neurons recorded in layer 2/3 of the GRSC. The left panel (black traces) shows the regular spiking firing found in some neurons; the right panel (red traces) shows the late spiking firing pattern. Insets: two superimposed action potentials shown on larger time scales. The black arrow in the right panel shows the slow subthreshold depolarization that characterized the late-spiking (LS) firing pattern. Scale bars on the right also apply to the left panel. **(C)** I/V relationship of non-LS (black symbols; *n* = 5–16) and LS pyramidal neurons (red symbols; *n* = 9–20). **(D)** firing frequency vs. injected current measured in non-LS (black symbols; *n* = 5–16) and LS pyramidal neurons (red symbols; *n* = 5–16). The firing was evoked by square depolarizing current pulses as shown in panel B; the firing frequency was averaged throughout the firing in both LS and non-LS neurons. **(E)** Neurolucida drawings of two LS (left) and two non-LS neurons (right).

**Figure 2:**
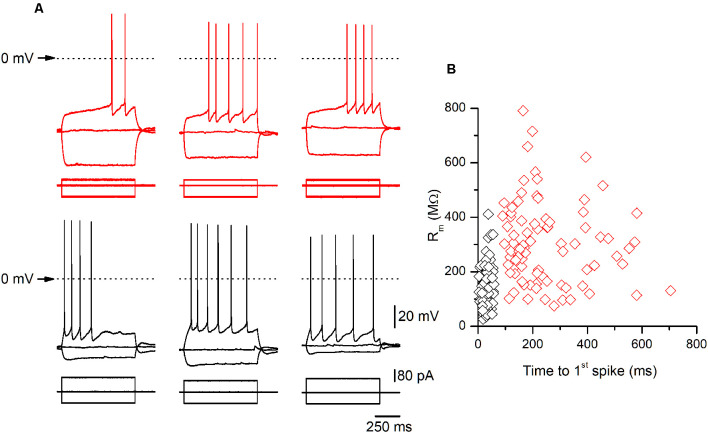
Examples of late-spiking (LS) and non-LS pyramidal neurons. **(A)** Examples of the responses to hyperpolarizing and depolarizing current pulses of three LS (upper panels, red traces) and three non-LS pyramidal neurons (lower panels, black traces). Note the clear subthreshold slow depolarization in LS neurons, which is absent in the non-LS neurons. The hyperpolarizing pulse was of the same magnitude in all neurons (−80 pA) to illustrate the difference in input resistance (IR) between both types of neurons. The depolarizing current pulse was +40 pA in LS neurons (threshold pulse strength) and +80 / +100 pA in the non-LS neurons (minimum pulse strength necessary to evoke tonic discharge). The dotted line marks the 0 mV level. The scale bars apply to all panels. **(B)** The relationship between the input membrane resistance and time to 1^st^ spike in LS (red symbols) and non-LS neurons (black symbols). The input resistance (R_m_) was measured from the responses to small hyperpolarizing current pulses, and the time to 1^st^ spike was measured between the onset of the depolarizing current pulse and the peak of the first spike fired by a depolarizing current pulse of strength corresponding to the rheobase.

**Figure 3:**
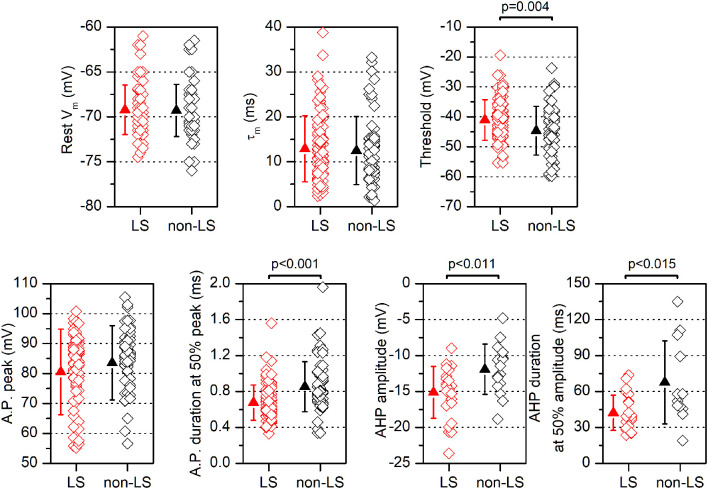
Electrophysiological parameters are measured in LS and non-LS neurons. Each panel shows the values obtained from individual neurons (diamonds) and the mean ± s.d. (triangles) of several parameters measured in LS neurons and non-LS neurons. The resting membrane potential (V_m_) was measured immediately after entering into the whole-cell mode; the threshold was measured at the 1^st^ action potential in a just-threshold response; the action potential duration was measured at half amplitude; the peak amplitude of the action potential after-hyperpolarization (AHP) was measured from the threshold level, and the AHP duration was measured from the peak to 50% of the peak amplitude.

**Figure 4:**
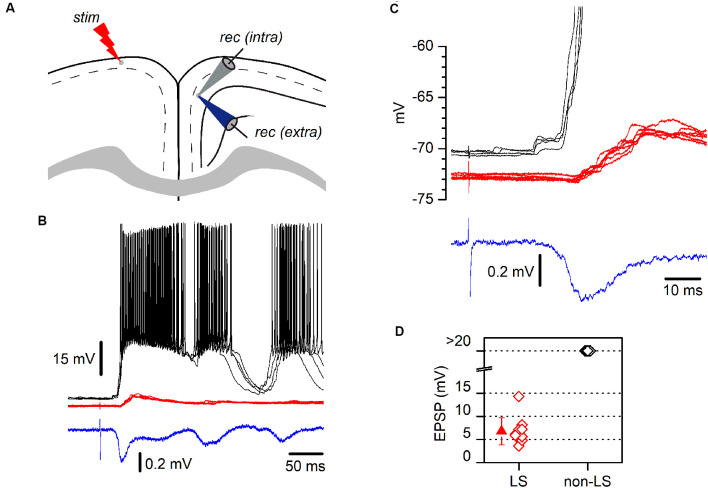
LS and non-LS pyramidal neurons respond differently to incoming epileptiform discharges. **(A)** The diagram of a coronal GRSC slice showing the positions of recording and stimulating electrodes. The extracellular recording electrode (blue electrode) was kept in the same position while successive neurons were recorded intracellularly (gray electrode); electrical stimuli were applied to the contralateral side (“stim”). **(B)** Responses of a non-LS (black traces) and an LS pyramidal neuron (red traces) during the propagation of epileptiform discharges (blue trace: extracellular recordings) evoked by contralateral stimulation. Both neurons were recorded sequentially and their somata were placed close together. Each panel features five consecutive superimposed traces. The extracellular recording was the average of five consecutive responses. **(C)** The initial section of the responses shown in panel B presented on larger time and voltage scales; note the delayed onset and smaller amplitude of the response recorded in the LS neuron (red traces). **(D)** The peak amplitude of the EPSPs recorded during the propagation of epileptiform discharges: LS neurons, red symbols; non-LS neurons, black symbols. When the response was supra-threshold, we assigned an amplitude of >20 mV.

### Statistics

Data are shown as values from individual neurons and/or giving the mean ± standard deviation (s.d.). Comparisons between two samples were made with non-parametric tests: the two-tailed Mann–Whitney rank-sum test for independent samples and the two-tailed Wilcoxon signed-rank test for paired samples. Comparisons of more than two samples (data on latencies shown in Figure 9) were made with the non-parametric Kruskal-Wallis one-way ANOVA on ranks which is independent of the samples’ distribution). If the ANOVA gave significant differences among groups (*p*-value < 0.05), a *post hoc* Dunn’s multiple comparison test was used to compare across all pairs of samples. Statistical analyses were performed using OriginPro8 (Origin Lab Corporation) or Sigma Stat 3.11 (Systat Software Inc). The degree of statistical significance is given in each figure and significance was set as *p* < 0.05.

**Figure 5:**
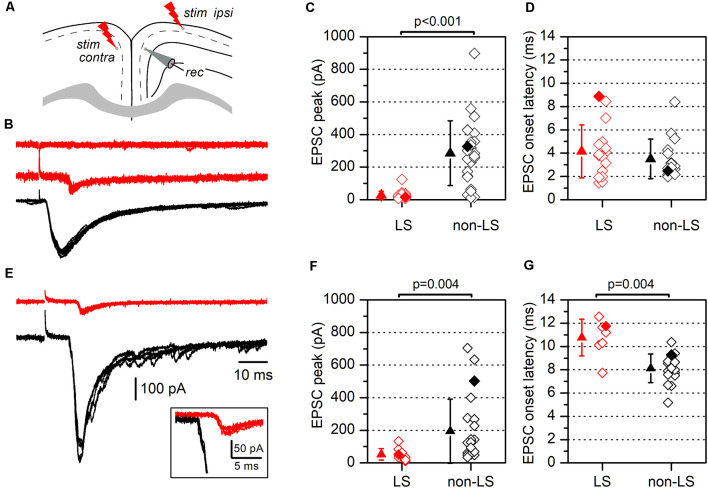
Synaptic currents evoked by electrical stimulation in LS and non-LS pyramidal neurons. **(A)** The diagram of a coronal slice from the GRSC showing the positions of recording and stimulation electrodes for ipsilateral and contralateral stimulation (“*stim ipsi*” and “*stim contra*”, respectively). **(B)** Examples of EPSCs recorded in response to ipsilateral electrical stimuli applied at 1.2 mm from the recorded neuron in one non-LS neuron (black traces) and two LS neurons (red traces). Each panel comprises four consecutive superimposed sweeps. **(C)** The peak amplitude, and **(D)** the latency of the EPSCs recorded in LS (red symbols; *n* = 20) and non-LS pyramidal neurons (black symbols; *n* = 25). **(E)** The examples of EPSCs recorded in response to contralateral stimulation in one non-LS (black traces) and one LS neuron (red traces). Each panel contains four consecutive superimposed sweeps. Note the longer latency of the response recorded in the LS neurons, shown at a larger scale in the inset. **(F)** The peak amplitude, and **(G)** the latency of the EPSCs evoked by contralateral stimulation. The peak amplitude and latency of the responses shown in panels **(B,E)** are highlighted by filled diamonds in panels **(C,D)** and **(F,G)**, respectively.

**Figure 6:**
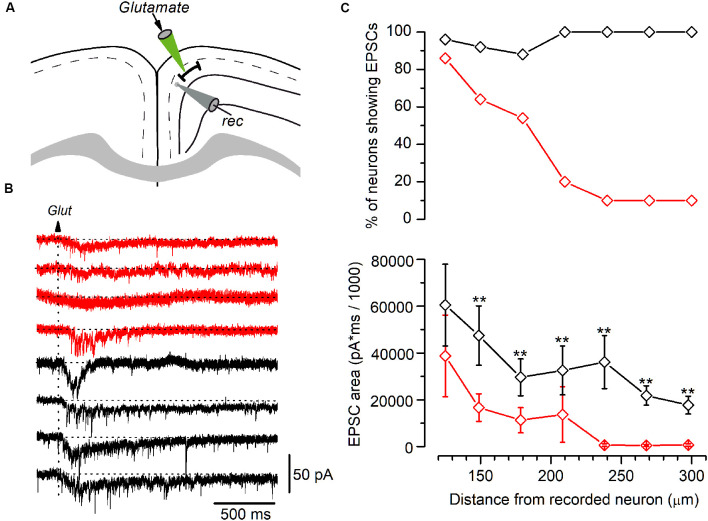
Synaptic currents elicited by glutamate application in LS and non-LS pyramidal neurons. **(A)** The diagram of a GRSC coronal slice showing the positions of the recording electrode (gray) and the pipette used to apply glutamate pressure puffs (green). **(B)** The synaptic currents elicited in four LS neurons (red traces) and four non-LS neurons (black traces) by a puff of glutamate applied at 180 μm from the soma of the recorded neurons (arrow). **(C)** The percent of neurons showing glutamate-evoked EPSCs (upper plot) and the average area of the EPSCs (lower plot) at different distances from the soma, where red symbols represent the LS neurons (125 μm: *n* = 28, show EPSCs *n* = 24; 150 μm: *n* = 28, show EPSCs *n* = 18; 180 μm: *n* = 28, show EPSCs *n* = 15; 210 μm: *n* = 10, show EPSCs *n* = 2; 240 μm: *n* = 10, show EPSCs *n* = 1; 270 μm: *n* = 10, show EPSCs *n* = 1; 300 μm: *n* = 10, show EPSCs *n* = 11), and black symbols the non-LS neurons (125 μm: *n* = 26, show EPSCs *n* = 25; 150 μm: *n* = 24, show EPSCs *n* = 22; 180 μm: *n* = 24, show EPSCs *n* = 21; 210 μm: *n* = 11, show EPSCs *n* = 11; 240 μm: *n* = 11, show EPSCs *n* = 11; 270 μm: *n* = 11, show EPSCs *n* = 11; 300 μm: *n* = 11, show EPSCs *n* = 11); **p < 0.01. At < 100 μm or shorter glutamate evoked large currents due to its direct effect on the recorded neurons’ somata. The area of the synaptic responses was measured by the integration of the current trace over time (1 s) from the baseline [horizontal dotted lines in panel **(B)**]; the baseline was calculated from the current average of the 200 ms previous to the glutamate application.

**Figure 7:**
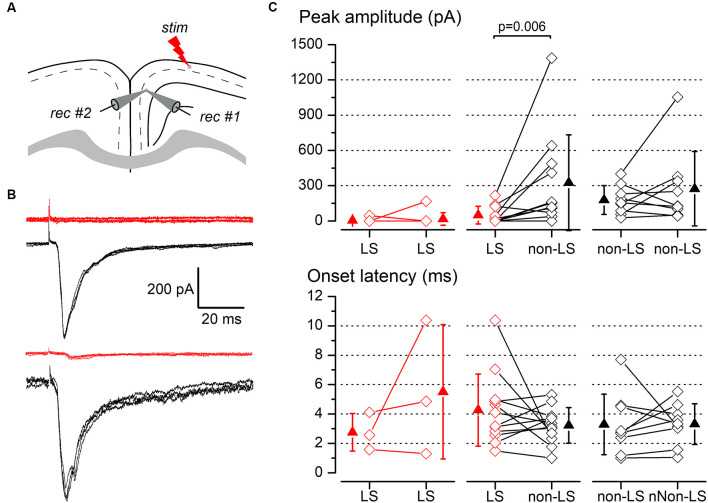
Synaptic responses to ipsilateral stimulation of simultaneously recorded pairs of layer 2/3 pyramidal neurons. **(A)** The diagram of a coronal slice of the GRSC showing the positions of the stimulus and recording electrodes. **(B)** Examples of synaptic responses recorded in two neuron pairs, each formed with one LS (red traces) and one non-LS neuron (black traces); in the upper pair, the LS neuron did not respond and in the lower pair, the LS showed a small EPSC. Each panel comprises five consecutive superimposed responses. **(C)** Peak amplitude (upper panels) and onset latency (lower panels) of the synaptic responses recorded in neuron pairs formed with two LS neurons (left panels), one LS and one non-LS neuron (middle panels), and two non-LS neurons (right panels). The diamonds show the data from individual neurons and the triangles show the mean ± s.d.

**Figure 8:**
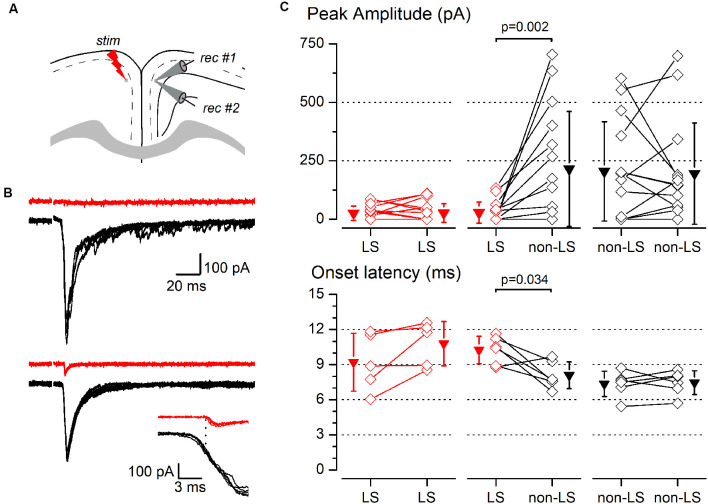
Synaptic responses to contralateral stimulation of simultaneously recorded pairs of layer 2/3 pyramidal neurons. **(A)** The diagram of a coronal slice of the GRSC showing the positions of the stimulus and recording electrodes. **(B)** Examples of synaptic responses recorded in two neuron pairs, each formed with one LS (red traces) and one non-LS neuron (black traces); in the upper pair, the LS neuron did not respond and in the lower pair, the LS showed a small EPSC. The inset shows the onset phase of the responses of the lower example plotted on a larger time scale to highlight the difference in the onset latencies. Each panel shows five consecutive superimposed responses. **(C)** Peak amplitude (upper panels) and onset latency (lower panels) of the synaptic responses recorded in neuron pairs formed by two LS neurons (left panels), one LS and one non-LS neuron (middle panels), and two non-LS neurons (right panels). Comparisons were made with the signed-rank test for paired samples. The diamonds show the data from individual neurons and the triangles show the mean ± s.d.

**Figure 9:**
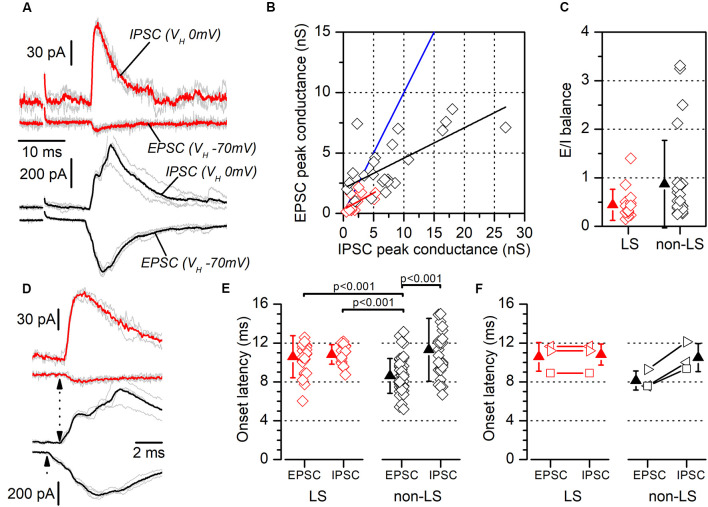
Excitatory/inhibitory balance and latency of responses evoked by contralateral stimuli. **(A)** Examples of recordings of EPSCs and feed-forward IPSCs evoked in a neuron pair formed with one LS neuron (red traces) and one non-LS neuron (black traces). The EPSCs were recorded at a holding potential of −70 mV and the IPSCs at 0 mV. Each panel shows five consecutive responses (gray traces) and their average (thick, red, or black traces). **(B)** EPSC peak conductance vs. IPSC peak conductance measured in individual neurons (LS, red; non-LS, black symbols). Linear fit to data from non-LS neurons: black line, slope: 0.251, R^2^: 0.44; linear fit to data from LS neurons: red line, slope: 0.277, R^2^: 0.29. The blue line is the identity line. **(C)** E/I balance measured in LS (red) and non-LS (black) neurons. **(D)** The rising phase of the synaptic responses for panel **(A)** plotted on an enlarged time scale to highlight the differences in the EPSC and IPSC onset latencies (dotted arrows). **(E)** EPSCs, and the IPSCs onset latencies recorded in LS (red symbols) and non-LS neurons (black symbols) neurons. For comparison with the IPSC latencies, in this figure, we have pooled the EPSC onset latencies recorded from individual recordings (data in Figure 5G) and paired recordings (data in Figure 8C). The onset latencies were compared using the Kruskal–Wallis one-way ANOVA on ranks and we used Dunn’s test for pairwise comparisons. **(F)** The onset latencies for the three neuron pairs formed with one LS and one non-LS neuron that both responded to contralateral stimuli. As in panel **(E)**, the EPSC onset latency data are taken from Figure 5G. In this panel each type of symbol represents the two neurons forming a simultaneously recorded pair.

## Results

### Presence of Pyramidal Neurons With a Late-Spiking Firing Pattern in the Layer 2/3 of the Mouse GRSC

All data were obtained from somatic whole-cell recordings made in pyramidal neurons whose somata were located in the most dorsal part of layer 2/3 of the GRSC (Figure 1A). We found that this cortical region had two types of pyramidal neurons, each with different electrophysiological properties and easily discernible from their responses to hyperpolarizing and depolarizing current pulses (Figure 1B). To characterize the electrophysiological properties of these neurons we made an initial set of experiments using protocols with different series of hyper- and depolarizing current pulses. Some pyramidal neurons (Figure 1B, left panel) showed a regular spiking pattern, similar to that described for layer 2/3 pyramidal neurons of the dysgranular RSC (Sempere-Ferràndez et al., 2018). However, other pyramidal neurons showed, in response to long depolarizing current pulses, a pronounced slow depolarizing ramp that led to the threshold and delayed spike firing (Figure 1B, right panel); this firing pattern was similar to the late spiking firing (LS) pattern described in the GRSC of the rat (Kurotani et al., 2013). This slow depolarization ramp causing the late spiking was quantified measuring the time from the start of the depolarizing current pulse to the peak of the first spike in response to a current pulse of a strength corresponding to the rheobase (time to 1^st^ spike). In pyramidal neurons showing this depolarization ramp the time to 1^st^ spike was always longer than 90 ms, and were classified as Late Spiking pyramidal neurons (LS neurons). The remaining neurons were classified as non-LS pyramidal neurons, and their time to 1^st^ spike was always shorter than 60 ms. According to our sample of pyramidal neurons, approximately 60% in the dorsal area of layer 2/3 of the GRSC were LS and 40% were non-LS, with the two types of neurons found to be readily intermingled. The LS neurons had a steeper I/V relationship (Figure 1C) than the non-LS neurons and fired at higher frequencies, revealing a non-saturating firing frequency vs. injected current relationship (Figure 1D). In response to suprathreshold current pulses, LS and non-LS neurons showed tonic firing, but with different degree of frequency adaptation. Frequency adaptation was measured as the quotient between the first and the last inter-spike interval in a current pulse of rheobase +40 pA; in LS neurons this quotient was close to 1 (no frequency adaptation), while in non-LS neurons was less than 1 (LS: 0.91 ± 0.26, *n* = 21; non-LS: 0.42 ± 0.18, *n* = 14; *p* < 0.001). In response to hyperpolarizing current pulses, non-LS neurons showed a clear voltage sag, which was smaller in LS neurons; this voltage sag was measured as the voltage difference between the initial hyperpolarization and the steady-state value at the end of a current pulse of −40 pA (LS neurons: 0.92 ± 0.80 mV, *n* = 21; non-LS neurons 4.16 ± 3.49 mV, *n* = 13; *p* = 0.002). LS neurons also had smaller somata when viewed in living slices under DIC optics (LS: 173 ± 56.59 μm^2^ *n* = 12, non-LS: 474.02 ± 119.86 μm^2^, *n* = 12; *p* < 0.001). A sample of neurons was stained intracellularly with biocytin (Figure 1E). Figure 2A shows several examples of LS and non-LS neurons to illustrate the firing pattern of LS and non-LS neurons. In addition to a shorter time to 1^st^ spike, LS neurons had a higher membrane input resistance (Figure 2B. Time to 1^st^ spike: LS 252.22 ± 139.84 ms, *n* = 90, non-LS: 32.36 ± 14.94 ms *n* = 61; *p* < 0.001; membrane input resistance: LS 310.72 ± 157.20 MΩ *n* = 90, non-LS 148.58 ± 81.39 MΩ *n* = 61, *p* < 0.001). Figure 2B also shows that values of the time to 1^st^ spike in LS and non-LS neurons did not overlap. The electrophysiological properties of these two types of pyramidal neurons are given in Figure 3. Several of these electrophysiological parameters were similar in both types of neurons (resting membrane potential: LS −69.22 ± 2.76 mV, *n* = 90, non-LS −69.29 ± 2.89 mV, *n* = 61; membrane time constant: LS 12.91 ± 7.33 ms *n* = 90, non-LS 12.48 ± 7.56 ms *n* = 61; action potential peak amplitude: LS 80.52 ± 14.30 mV *n* = 90, non-LS 83.56 ± 12.40 *n* = 61), but others showed differences between LS and non-LS neurons (threshold for spike firing: LS −41.02 ± 6.77 mV *n* = 90, non-LS −44.62 ± 8.10 mV *n* = 61, *p* = 0.004; action potential duration: LS 0.68 ± 0.20 ms *n* = 90, non-LS 0.85 ± 0.28 ms *n* = 61, *p* < 0.001; AHP amplitude: LS −15.12 ± 3.59 mV *n* = 24, non-LS −11.92 ± 3.49 mV *n* = 16, *p* < 0.011; AHP duration: LS 42.28 ± 14.69 ms *n* = 22, non-LS 67.66 ± 34.64 ms *n* = 12, *p* < 0.015). Not all parameters could be measured reliably in each neuron. The higher input resistance of LS neurons together with a similar membrane time constant suggests that the LS neurons had a lower membrane capacitance, which is consistent with a smaller size.

### LS and Non-LS Pyramidal Neurons Behave Differently During the Propagation of Epileptiform Discharges

LS and non-LS pyramidal neurons were readily intermingled in the dorsal part of the GRSC; however, despite their proximity, they responded very differently during the propagation of epileptiform discharges along with the RSC (Figure 4). Epileptiform discharges were evoked by stimulation of layer 1 in the presence of a GABA_A_ blocker (bicuculline 10 μM) and a modified ACSF (see “Materials and Methods” section). Under these conditions, large epileptiform discharges are known to propagate from the stimulation point to the ipsilateral layer 2/3 and, through the corpus callosum, to the contralateral cortex (Rovira and Geijo-Barrientos, 2016). All non-LS neurons recorded during the propagation of epileptiform discharges showed large depolarizations which always reached the threshold and provoked action potential burst firing (Figures 4B,C); in fact, the discharges recorded extracellularly were caused by the firing of non-LS neurons. Contrastingly, all recorded LS neurons presented only small, and always subthreshold, polysynaptic EPSPs (peak amplitude: 6.8 ± 2.94 mV, *n* = 10; Figure 4D). These synaptic responses often appeared delayed concerning the responses in non-LS neurons (Figures 4B,C). We do not know the circuit mechanisms responsible for the generation of the synaptic responses recorded during the propagation of epileptiform discharges, but the different responsiveness during this type of activity suggests that LS and non-LS pyramidal neurons form part of different neuronal circuits within layer 2/3, although they are located in very close to each other within the dorsal part of layer 2/3. These data suggest that propagating epileptiform seizures were only supported by non-LS neurons and that LS neurons were not involved in this kind of activity.

### Long-Range Cortico-Cortical Axons Mostly Innervate Non-LS Pyramidal Neurons

One explanation of the different responses from LS and non-LS neurons during the propagation of epileptiform discharges is that they receive different afferent innervation. To test this possibility, we studied the synaptic currents evoked by stimulating long-range cortico-cortical axons. These axons were stimulated by applying electrical stimuli to two different sites: the ipsilateral layer 2/3 at 1.2 mm from the recording area and the homotopic contralateral layer 2/3 (Figure 5); the ipsilateral stimulation site was located in the dysgranular RSC. In response to ipsilateral layer 2/3 supra-maximal stimulation (Figures 5B,C), non-LS neurons generated large EPSCs, while LS neurons did not respond or generated only small EPSCs (non-LS: 285.39 ± 198.53 pA, *n* = 25; LS: 27.35 ± 24.66 pA, *n* = 20; *p* < 0.001). Similar results were observed after stimulation of the contralateral cortex (Figures 5E,F): non-LS neurons generated large EPSCs but LS neurons produced significantly smaller EPSCs (non-LS: 194.0 ± 196.63 pA, *n* = 22; LS: 51.91 ± 35.26 pA, *n* = 10; *p* = 0.004). The EPSCs recorded in the LS neurons had longer average latencies than the non-LS neurons in response to contralateral stimulation (Figure 5G; non-LS: 8.13 ± 1.23 ms, *n* = 17; LS: 10.76 ± 1.58 ms, *n* = 7; *p* = 0.004), but not in response to ipsilateral stimulation (Figure 5D; non-LS: 3.50 ± 1.70 ms, *n* = 16; LS: 4.15 ± 2.29 ms, *n* = 16).

We also measured the rise and decay times of the EPSCs evoked by contralateral stimulation. The rise time was significantly shorter in LS neurons (1.45 ± 0.45 ms, *n* = 12 vs. 3.27 ± 0.68 ms in non-LS neurons, *n* = 9; *p* < 0.001), while the decay time (measured as the time constant for a single exponential fit) was similar in both types of neurons (5.58 ± 2.15 ms in LS, *n* = 12 vs. 7.54 ± 2.53 ms in non-LS neurons, *n* = 9; *p* = 0.07). For the measurement of the EPSC time course in non-LS neurons, we selected cells in which the synaptic current did not show delayed di- or polysynaptic responses to make possible the fitting of the decay phase to a single exponential. In this sample of neurons, the peak amplitude was smaller in LS compared to non-LS neurons (50.12 ± 41.72 pA, *n* = 12; 218.11 ± 186.53 pA, *n* = 9; *p* = 0.021). The different magnitude of the synaptic responses correlated with the very different probabilities of the firing action potentials in response to ipsilateral or contralateral stimuli. Before going into the voltage-clamp mode, we checked whether electrical stimuli were able to evoke suprathreshold responses and action potential firing in a sample of neurons. We found that ipsilateral stimuli caused action potential to fire in 0 out of 30 (0%) LS and 12 out of 15 (80%) non-LS neurons, whereas with contralateral stimuli, action potentials fired in 0 out of 31 (0%) LS neurons and 5 out of 43 (12%) non-LS neurons.

Our results suggest that LS neurons receive less excitatory synaptic contacts that originated in long-range cortico-cortical axons. For a more accurate estimate of the range over which LS and non-LS neurons receive afferents, we studied the synaptic responses evoked in both types of neurons by the local glutamate application. Glutamate was applied using pressure puffs by placing the tip of patch electrodes at different distances from the recorded neurons. This approach allowed us to activate neurons close to the tip of the glutamate pipette and therefore determine the maximum distance from which both types of neurons received excitatory axons, as shown in Figure 6. For each recorded neuron we explored the synaptic responses evoked by applying glutamate on layer 2/3 at 100–300 μm from the soma of the recorded neuron. Synaptic responses were recorded within this range in 88–100% of non-LS neurons; however, they were mostly recorded when glutamate was applied at distances of less than 200 μm in LS neurons (54–86% of the LS neurons). The number of LS neurons that responded to glutamate decreased drastically when glutamate was applied at distances of more than 200 μm (20% to 10% of the LS neurons). These data showed that the LS neurons were innervated by axons originated in nearby layer 2/3 neurons (within 250 μm); in contrast, non-LS neurons receive axons from layer 2/3 neurons located further away.

The above data were recorded and pooled from neurons individually recorded in different slices. Given that the stimulating electrode was not always placed in the same position in different slices and the efficacy of the electrical stimuli varied, the pooling of data from different slices introduced a variability factor that could affect the results, including the differences in the latencies of the evoked responses (Figure 5E). Also, in some neurons of this set of experiments the latency could not be measured reliably. To confirm our results and eliminate any variability introduced by differences in the stimuli among slices, we conducted a series of experiments based on the simultaneous recording of pairs of neurons. The position of the stimulating electrode and the stimulus strengths were exactly the same for both neurons in each pair of recorded neurons. The distance between the somata of simultaneously recorded neuron pairs was 109.23 ± 81.08 μm (range 20–320 μm; *n* = 78). All recorded neuron pairs were checked for synaptic connection between the neurons forming the pair. In a total of 78 pairs recorded (28 formed by LS—LS neurons, 27 by LS—non-LS neurons, and 23 by non-LS—non-LS neurons) we detected only six cases of synaptic connections; five were from LS to non-LS and one was from non-LS to non-LS (this latter connection was only unidirectional). In response to either long-range ipsilateral stimulation (Figure 7) or contralateral stimulation (Figure 8), we found that LS neurons received excitatory inputs of significantly smaller amplitudes when comparing neurons from pairs formed by a non-LS and an LS neuron (ipsilateral stimulation: non-LS, 325.02 ± 406.88 pA; LS, 49.26 ± 75.28 pA; *n* = 11 pairs; *p* = 0.006. Contralateral stimulation: non-LS, 215.05 ± 245.63 pA; LS, 28.68 ± 44.81 pA; *n* = 15 pairs; *p* = 0.002). In homogeneous pairs, there were no differences in the EPSC peak amplitude of both neurons in response to ipsilateral or contralateral stimuli (Ipsilateral stimuli: LS/LS pairs 4.52 ± 14.29 pA vs. 16.74 ± 52.93 pA, *n* = 10 pairs; non-LS/non-LS pairs: 178.44 ± 121.74 pA vs. 273.87 ± 316.37 pA, *n* = 9 pairs. Contralateral stimulation: LS/LS pairs 26.04 ± 30.20 pA vs. 26.64 ± 40.49 pA, *n* = 18 pairs; non-LS/non-LS pairs: 205.16 ± 2,012.07 pA vs. 195.66 ± 216.15 pA, *n* = 14 pairs). The EPSCs recorded in response to contralateral stimulation had longer latencies in LS neurons (Figure 8C) when comparing the latencies in LS/non-LS pairs (LS: 10.25 ± 1.18 ms; non-LS: 8.09 ± 1.14 ms *n* = 6 pairs; *p* = 0.034); however, the EPSCs recorded in response to ipsilateral stimulation in LS neurons also had, on average, longer latencies, but the difference was not statistically significant (Figure 7C; LS: 4.27 ± 2.45 ms, non-LS: 3.2 3 ± 1.21 ms, *n* = 11 pairs). In LS/LS and non-LS/non-LS pairs, there were no differences between the latencies of the neurons forming a pair, either in response to ipsilateral (LS/LS pairs: 2.75 ± 1.27 ms vs. 5.51 ± 4.58 ms, *n* = 3 pairs; non-LS/non-LS pairs: 3.29 ± 2.06 ms vs. 3.32 ± 1.39 ms, *n* = 9 pairs) or contralateral stimulation (LS/LS pairs: 9.21 ± 2.48 ms vs. 10.79 ± 1.90 ms, *n* = 5 pairs; non-LS/non-LS pairs: 7.35 ± 1.08 ms vs. 7.45 ± 1.02 ms, *n* = 6 pairs).

### Excitatory/Inhibitory Balance Was Similar in LS and Non-LS Pyramidal Neurons

Another mechanism that could contribute to the different size of the synaptic currents is a smaller excitatory/inhibitory balance (E/I balance) in LS compared to non-LS neurons. Neuronal microcircuits in superficial cortical layers are characterized by a powerful feed-forward inhibitory system arising from parvalbumin-expressing fast-spiking GABAergic interneurons (Holmgren et al., 2003; Avermann et al., 2012). As in the dysgranular RSC (Sempere-Ferràndez et al., 2019), the activation of afferent axons in layer 2/3 of the GRSC evoked a large feed-forward inhibitory component, in both LS and non-LS pyramidal neurons. The presence of this feed-forward inhibition means the net action of afferent axons on the postsynaptic neurons depends on the balance between the direct excitatory input and the feed-forward inhibition. In GRSC pyramidal neurons the E/I balance was measured from the excitatory and inhibitory conductance of the synaptic responses evoked by ipsilateral or contralateral stimulation in a sample of LS and non-LS neurons (Figure 9). Excitatory conductance was measured at the peak of the EPSCs recorded at −70 mV (a value close to the chloride equilibrium potential under our experimental conditions), while the inhibitory conductance was measured at the peak of the IPSCs recorded at 0 mV (a potential close to the AMPA receptors reversal potential of). The E/I balance was generally <1 in both LS and non-LS neurons (Figure 9C; LS: 0.44 ± 0.32, *n* = 15; non-LS: 0.87 ± 0.90, *n* = 25) and there was no difference in E/I balance between LS and non-LS neurons (Figure 9C). This result confirms that differences in the E/I balance were not responsible for the different magnitudes observed in the synaptic responses evoked by long-range axons in LS and non-LS neurons.

### The Excitatory Responses to Contralateral Stimuli Recorded in LS Neurons Were Disynaptic

We measured the latencies of the EPSCs and IPSCs evoked by contralateral stimuli (Figures 9E,F; examples shown in Figures 9A,D). In LS neurons, EPSCs and IPSCs latencies were 10.58 ± 2.16 ms (*n* = 22) and 10.82 ± 1.00 ms (*n* = 18), respectively; and in non-LS neurons they were: 8.62 ± 1.79 ms (*n* = 47) in EPSCs and 11.30 ± 3.23 ms (*n* = 31) in IPSCs. These differences were also apparent in simultaneously recorded neuron pairs (*n* = 3; Figure 9F), with an EPSC of 10.59 ± 1.48 ms, and an IPSC of 10.81 ± 1.09 ms in LS neurons, compared to an EPSC of 8.14 ± 0.99 ms, and an IPSC of 10.49 ± 1.45 ms in non-LS neurons. The latencies were compared with the Kruskal–Wallis ANOVA on ranks, using Dunn’s test for pairwise comparisons (Figure 9E). This analysis showed that the latencies of the EPSCs recorded in non-LS neurons were significantly shorter than the latencies measured for the IPSCs in non-LS neurons and the EPSCs and IPSCs from LS neurons. These values suggest that the EPSCs recorded in non-LS neurons were monosynaptic, while the EPSCs recorded in LS neurons (feed-forward excitation) and the IPSCs recorded in both neuron types were disynaptic since the average latency of the latter group of responses was 2.7–3 ms longer than that of the non-LS EPSCs. What is more, the disynaptic nature of the IPSCs is consistent with their feed-forward mechanisms.

## Discussion

In this work, we have studied the electrophysiological properties of layer 2/3 pyramidal neurons in the mouse GRSC, as well as their synaptic responses to cortico-cortical axons. We have identified the presence of two electrophysiological types of pyramidal neurons in the dorsal part of this cortical area. Some neurons, about 60% of the whole sample, showed a prominent late-spiking firing pattern similar to that of layer 2/3 pyramidal neurons in the rat GRSC (Kurotani et al., 2013), while the remaining neurons had a regular spiking pattern that was very similar to that of layer 2/3 pyramidal neurons located in other cortical areas, including the dysgranular RSC (area 30; Sempere-Ferràndez et al., 2018). Our data from simultaneous paired recordings show that the LS and non-LS pyramidal neurons were readily intermingled in the dorsal GRSC, as we often found neuron pairs formed by an LS and a non-LS neuron whose somata were separated by 100 μm on average. This neuron mixture suggests that the dorsal portion of the GRSC is an area of transition between the more ventral GRSC (where layer 2/3 comprises almost exclusively LS neurons: 94% in the rat GRSC according to Kurotani et al., 2013) and the dorsally located dysgranular (which contains only regular spiking pyramidal neurons: Sempere-Ferràndez et al., 2018). However, we do not have further data supporting the hypothesis that the dorsal part of the GRSC is a transition area with the dysgranular RSC.

We have shown that the LS and non-LS pyramidal neurons were integrated with different neuronal circuits, even though these two types of neurons were found in proximity. Our data on glutamate application and the stimulation of long-range cortico-cortical axons (of either ipsilateral or contralateral origin) show that non-LS neurons receive cortico-cortical synaptic contacts from both local and long-range origin (over 250 μm) that generate complex EPSCs with mono- and polysynaptic components (see examples in Figures 5, 7); however, LS neurons were, by contrast, innervated mainly by cortico-cortical axons of local origin, but not by long-range axons. This finding is reinforced by the results for the synaptic responses evoked by the stimulation of long-range cortico-cortical axons, either ipsilaterally or contralaterally. The large difference in the magnitudes of the EPSCs and the proximity of LS and non-LS neurons implies that non-LS pyramidal neurons are selectively innervated by incoming long-range axons acting on layer 2/3 of the dorsal part of the GRSC. This selective long-range axonal innervation of non-LS neurons implies that LS and non-LS pyramidal neurons are integrated with different neuronal circuits, and therefore participate in different functions. The non-LS neurons fired intensely during epileptiform discharges evoked in the dis-inhibited cortex, while LS neurons only showed subthreshold synaptic responses.

An alternative explanation of the different magnitudes of the synaptic responses recorded in LS and non-LS neurons is that afferents originated in long-range axons were similar for both types of neurons, but the somatodendritic localization differed. In the LS neurons, the synapsis could be located in distal dendrites; whereas in the non-LS neurons, the synapsis could be located in proximal dendrites. It has been shown in thick tufted layer 5 pyramidal neurons that synaptic contacts formed on the apical dendrite do not correspond to the distance from the soma and therefore distal contacts generate smaller somatic synaptic responses than proximal contacts (Williams and Stuart, 2002). We cannot rule out this explanation, but the values of the rise and decay times of the EPSCs from LS and non-LS neurons are not fully consistent with different synaptic localization. Decay times were similar in both types of neurons, while rise times were even shorter for LS neurons. However, it is important to note that the EPSCs had polysynaptic components that may lengthen their time course in non-LS neurons.

Neurons with a late-spiking firing pattern have been found in other cortical areas of rodents related to the RSC and implicated in spatial information processing. Pyramidal-like neurons with a clear late-spiking pattern have been reported in the superficial layers of the presubiculum (Abbasi and Kumar, 2013), which is reciprocally connected to the GRSC (Wyss and Van Groen, 1992). In the medial entorhinal cortex, which receives connections from the RSC (Wyss and Van Groen, 1992), layer 2 non-stellate pyramidal-like neurons have also shown an LS pattern (Alonso and Klink, 1993). Finally, small, layer 2/3 pyramidal neurons with an LS firing pattern have been described in the perirhinal cortex (Faulkner and Brown, 1999); there are also LS neurons in layer 6 of this cortical region, but they are not pyramidal. Given that neurons with an LS firing pattern could be involved in the integration of responses with different timing (Kurotani et al., 2013), these neurons may form part of neural circuits equipped with specific signal processing timing capacities.

The characteristics of the synaptic responses evoked in LS and non-LS neurons by long-range cortico-cortical axons show that in the generation of these responses participated complex local neuronal circuits. The values of the onset latencies of the synaptic responses (originated in the contralateral cortex; Figure 9) suggests that in LS neurons the EPSCs were disynaptic, while in non-LS neurons the onset of the EPSCs was monosynaptic. On the other hand, in LS neurons the EPSCs were mostly small and had a single component (disynaptic), while in non-LS neurons the EPSCs were mostly large and complex, having an initial monosynaptic component and several delayed polysynaptic components (see recordings shown in Figures 5, 7). The simplest explanation for these findings is that long-range cortico-cortical axons reaching the dorsal part of the GRSC innervated monosynaptically only non-LS neurons; part of these non-LS neurons should fire action potentials and this firing generated the disynaptic EPSCs in LS neurons and the polysynaptic components in non-LS neurons by a feed-forward excitation. This hypothesis is supported by our finding that some non-LS neurons fire action potentials in response to long-range (80–12% of the recorded non-LS neurons in response to ipsi- and contralateral axons, respectively) and by the probability of interconnections between layer 2/3 pyramidal neurons (0.1–0.15, Holmgren et al., 2003; Avermann et al., 2012). However, in our sample of simultaneously recorded neuron pairs, we did not find a single case of non-LS to LS synaptic connections, which suggests that neurons causing the disynaptic responses in LS neurons were placed outside the dorsal part of the GRSC. Overall, these data show the complexity of the local neuronal circuits causing the synaptic responses to cortico-cortical axons in the dorsal part of the GRSC.

Our results may have two limitations associated with the method of recording the synaptic responses. Firstly, concerning the long-range stimulation experiments, the recorded responses (or part of them) may be due to local collaterals from neighboring pyramidal neurons activated antidromically by the electrical stimuli rather than the long-range afferent axons impinging on layer 2/3 neurons. This is particularly important in terms of the contralateral stimulation, given the symmetrical, bilateral structure of the callosal fibers. However, we did not record a single case of antidromic activation in response to contralateral stimulation throughout our entire sample of both LS and non-LS neurons. This observation means it is very unlikely that the synaptic responses recorded after contralateral stimulation could be caused by antidromic activation of the local collateral branches of the neuronal axons. This lack of any antidromic responses contrasted with the dysgranular RSC, where a very small proportion of layer 2/3 pyramidal neurons fire antidromically (<10% with maximal stimuli, Sempere-Ferràndez et al., 2018). Secondly, we used an intracellular solution based on K^+^-gluconate for voltage-clamp recordings, instead of a cesium-based solution. This was because it was impossible to identify LS and non-LS pyramidal neurons with intracellular cesium, given that the firing pattern changes drastically. However, as mentioned previously, the EPSC time course data rule out that small responses recorded in the LS neurons were originated in more distal dendrites than large responses recorded in the non-LS neurons, thus minimizing the relevance of using intracellular cesium.

Our results were obtained in mice aged 14–22 days. At this age, the cortical circuit is still not fully developed and, therefore, we cannot rule out the possibility that further circuit refinement may contribute to the mechanisms controlling the firing of pyramidal neurons and the different coding strategies of layer 2/3 and layer 5 pyramidal neurons. For instance, Angulo et al. (1999) showed that from weeks 3–5 postpartum some changes occur in the excitatory connections from layer 5 pyramidal neurons to fast-spiking interneurons, in particular, a switch from paired-pulse depression to paired-pulse facilitation that confers layer 5 pyramidal neurons wider integrative capabilities at 5 weeks of postnatal age. The fact that cortical circuits are not fully developed at this postnatal development stage means the afferent connections formed on LS and non-LS pyramidal neurons are still susceptible to subsequent refinements.

The innervation of the dorsal dysgranular RSC by callosal axons is denser than the innervation of the GRSC (Sempere-Ferràndez et al., 2018), and this is coincident with the different innervation by contralateral cortico-cortical axons between LS and non-LS neurons that we report here. From a functional point of view, this finding suggests that non-LS neurons could be part of the dorsal dysgranular RSC (their properties were very similar to those of the pyramidal neurons of this area, Sempere-Ferràndez et al., 2018), although they are placed within the morphologically defined GRSC. This would imply that the differences in synaptic responses found between LS and non-LS neurons could represent differences in microcircuit organization between the dorsal dysgranular RSC and the ventral GRSC.

## Data Availability Statement

The raw data supporting the conclusions of this article will be made available by the authors, under a reasonable request.

## Ethics Statement

The animal study was reviewed and approved by Ethical Committee for the Experimental Research of the Universidad Miguel Hernández.

## Author Contributions

RR, SM, and EG-B designed the research. RR, ED-S, and EG-B performed the research. RR and EG-B wrote the manuscript and all the authors revised it. All authors contributed to the article and approved the submitted version.

## Conflict of Interest

The authors declare that the research was conducted in the absence of any commercial or financial relationships that could be construed as a potential conflict of interest.
